# Supplementary material for: Haemato-Immunological Response of Immunized Atlantic Salmon (Salmo salar) to Moritella viscosa Challenge and Antigens
Source: Vaccines (Basel). 2024 Jan 10;12(1):70. doi: 10.3390/vaccines12010070 (PMC10818610; doi:10.3390/vaccines12010070)
Supplement: Supplementary file 1 [file vaccines-12-00070-s001.zip › vaccines-2715364-supplementary.pdf]

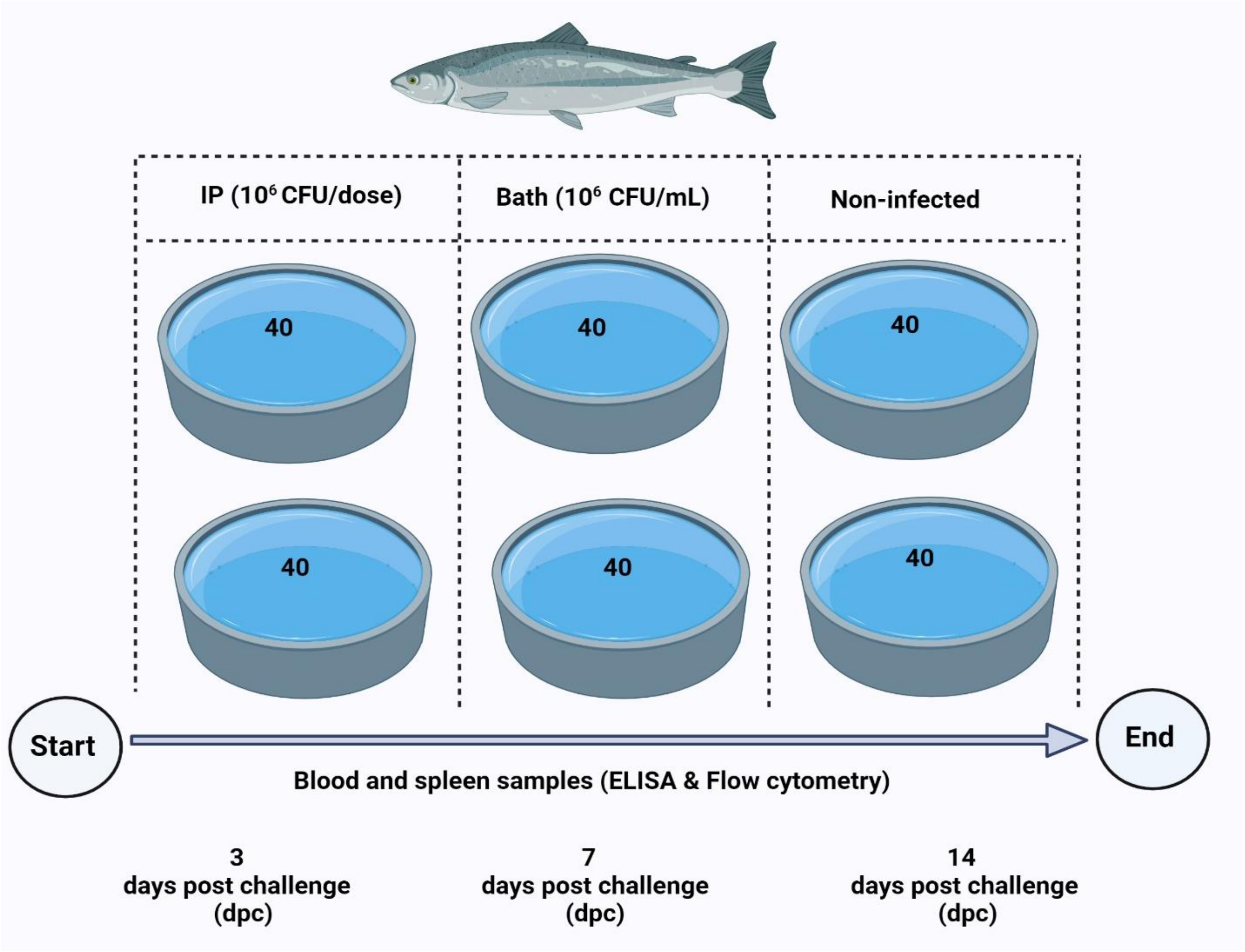

Figure S1. Challenge assay with *Moritella viscosa.* Blood and spleen samples were collected at 3, 7 and 14 days post challenge (dpc).

A

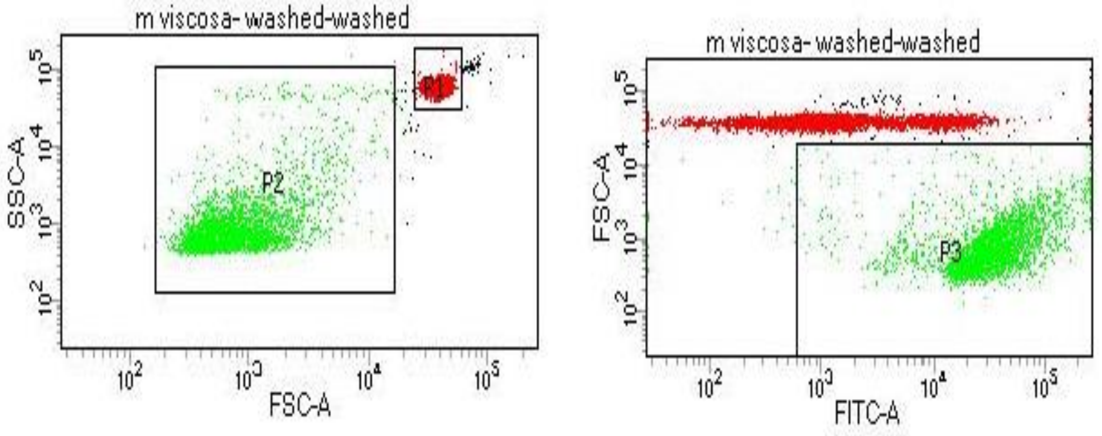

B

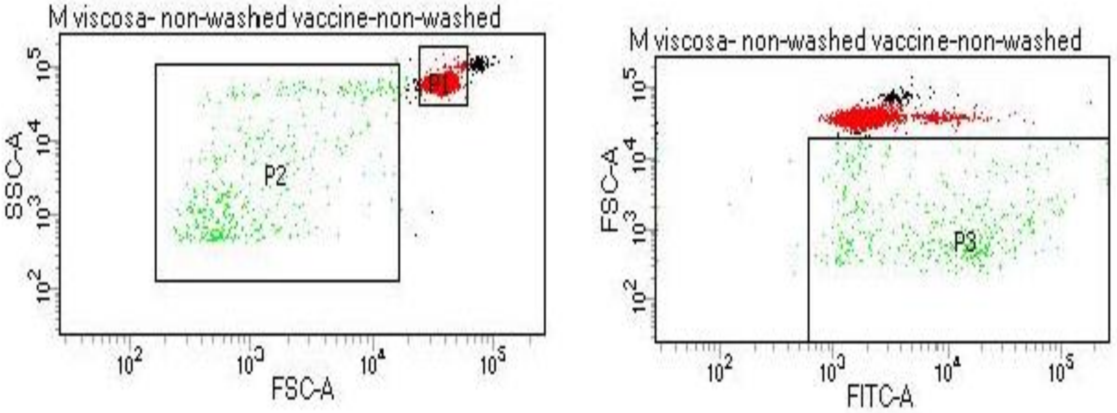

Figure S2. Vaccine concentration. The concentration of **(A)** bacterin cell and **(B)** complete bacterin vaccines were calculated based on the defined formula by flow cytometry (bacterial counting cell: (No of bacterial cell ($p2$))/ (No of beads ($p1$)) $\times 10^6 \times$ dilution (10^3) in flow cytometry.

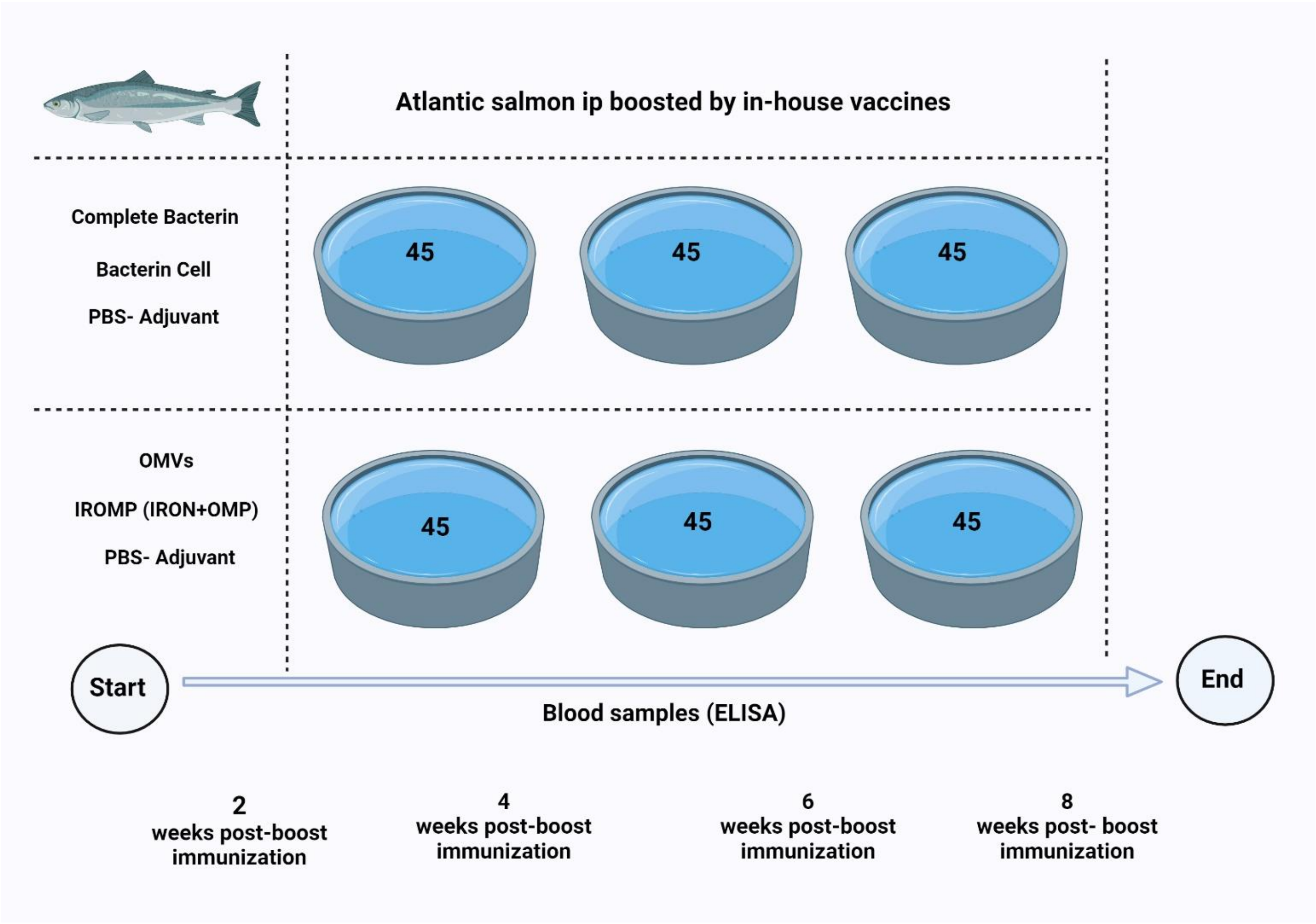

Figure S3. Post-boost immunization design by in house vaccines. Blood samples were collected at 2, 4, 6 and 8 weeks following the boost.

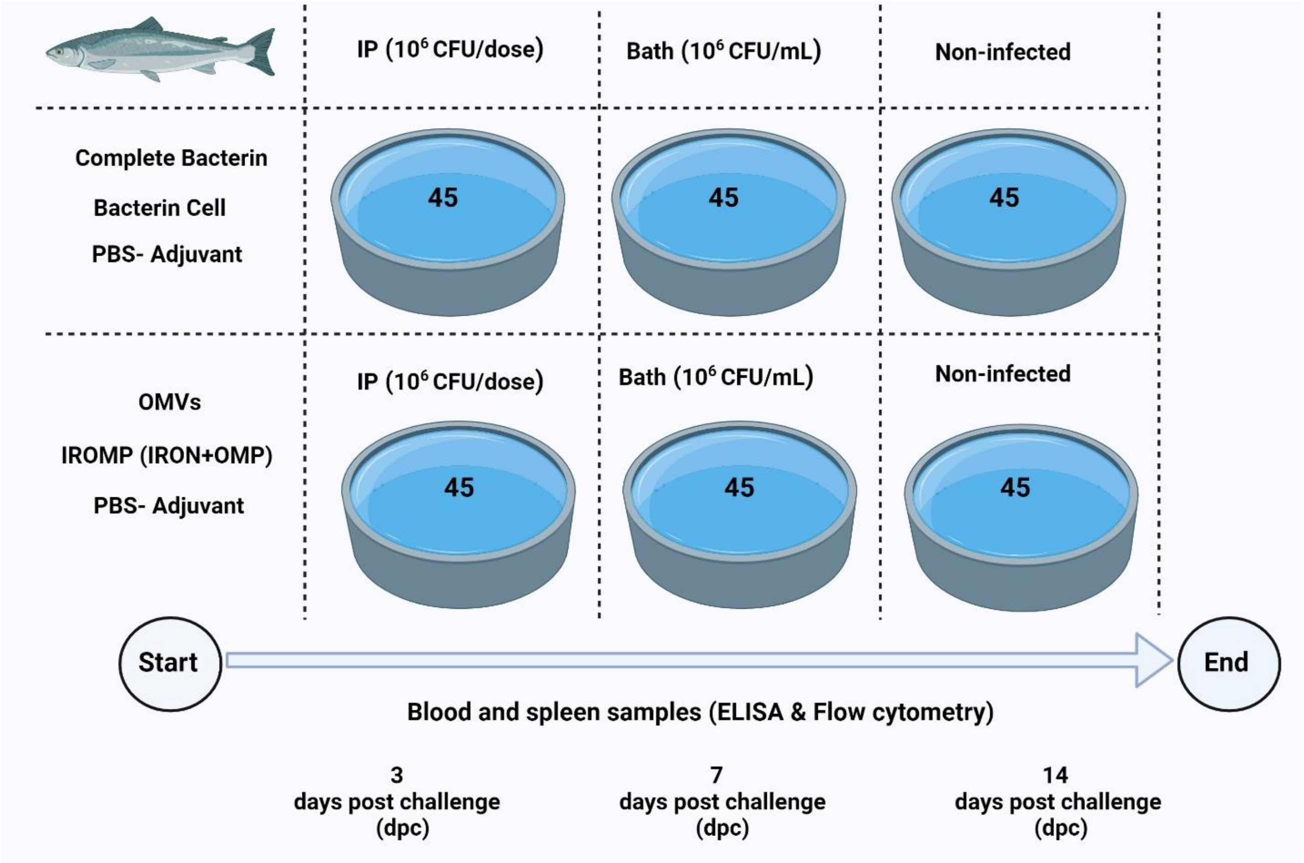

Figure S4. Challenge assay by *M. viscosa* at 12 weeks post boost immunization. Blood and spleen samples were collected at 3, 7 and 14 days post challenge (dpc).

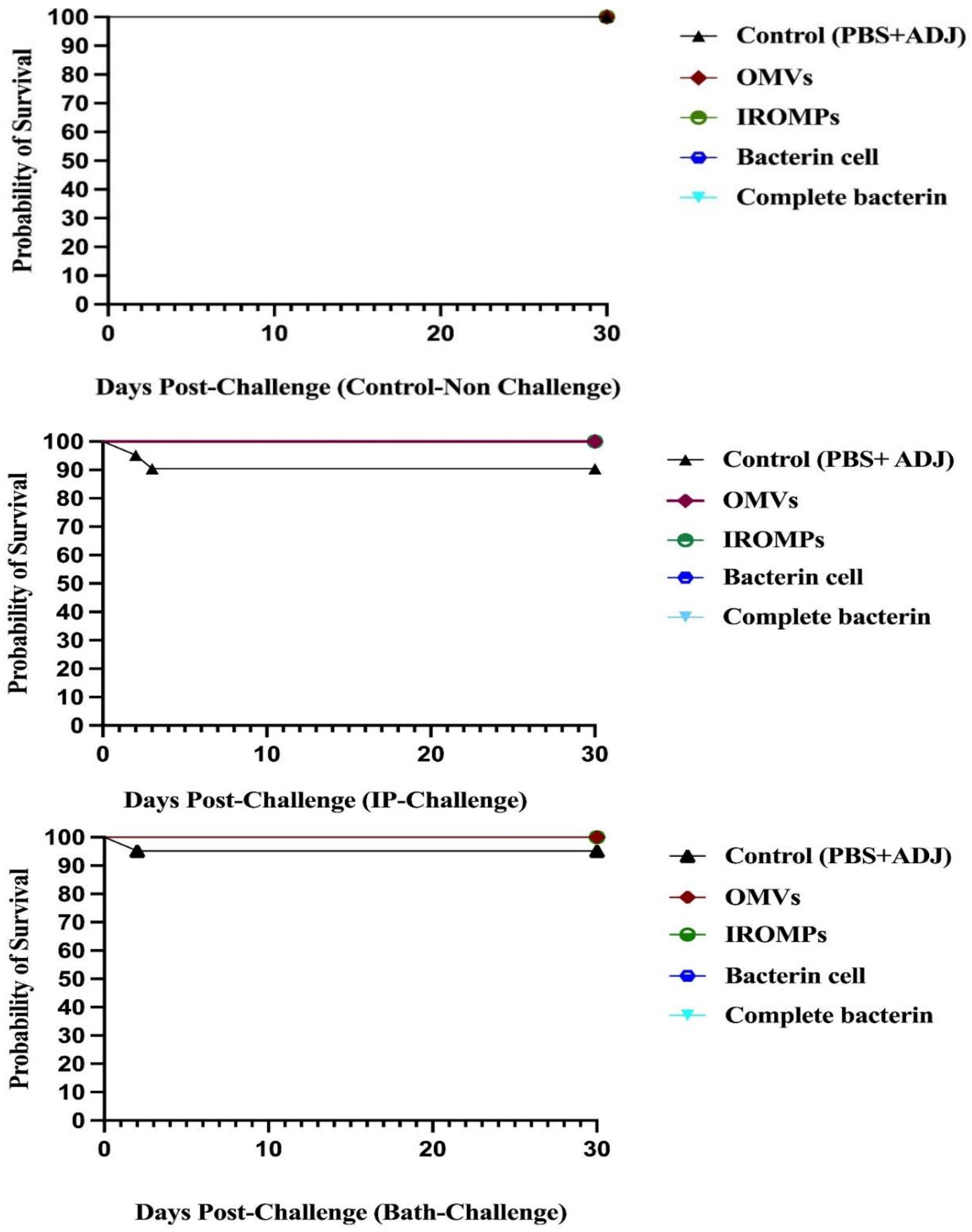

Figure S5. Survival proportions were compared between boosted and non-boosted (PBS+ADJ) Atlantic salmon after ip and bath challenges with *M. viscosa*. A group of animals was not subjected to any challenges.

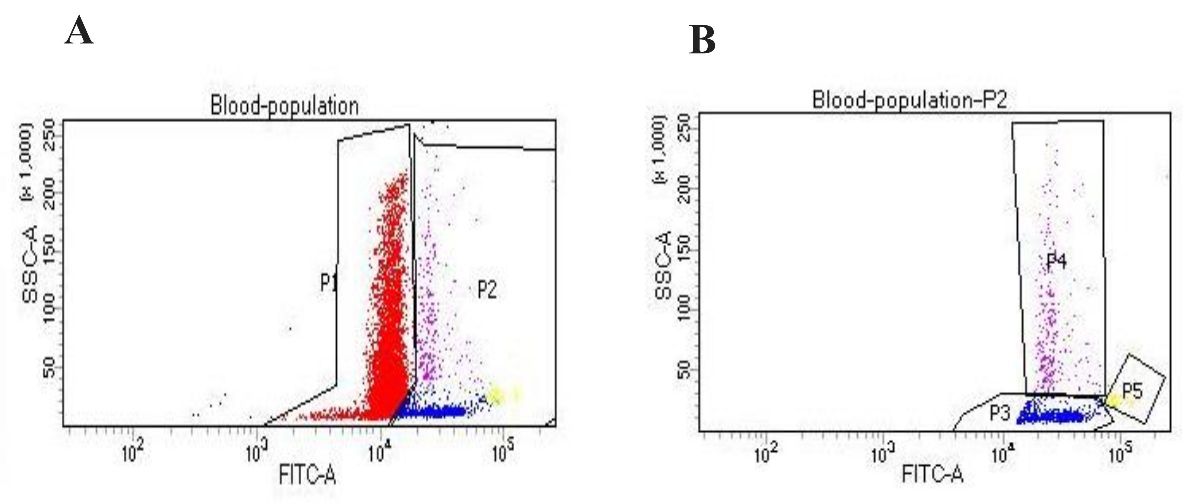

Figure S6. The flow cytometry algorithm for dividing blood populations. **(A)** P1 shows red blood cells (RBCs) and P2 shows white blood cells (WBCs) in vaccinated farmed Atlantic salmon. **(B)** P3 shows lymphocytes. P4 indicates neutrophils and basophils and P5 displays monocytes. The value of threshold operator was 5000 for FSC and SSC in flow cytometry. The voltages for FSC, SCC and FITC were 247, 231, and 313 respectively.

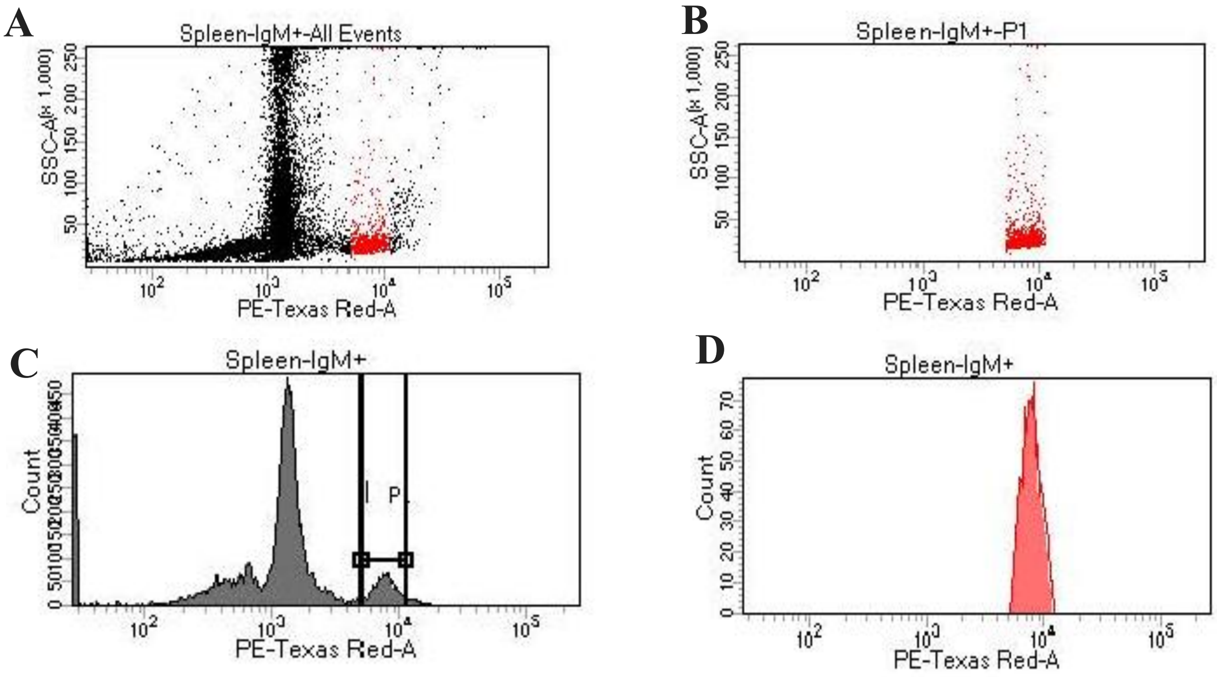

Figure S7. The flow cytometry technique for defining IgM+ (B cells) in spleen. **(A)** The red color displays IgM in the spleen. **(B)** Texas red is a marker to show IgM+ population. **(C)** Based on the DIOC6 the population of RBC and WBCs were separated. (**D)** The peak demonstrates the IgM+.
